# Contemporary Theory and Research

**Published:** 1893-07-29

**Authors:** 


### Contemporary Theory and Research.

### SIGNS OF DEATH.

M. Bourneville, in the *Progrès Médical*, calls attention to the importance of the thermometer in distinguishing real from apparent death. He cites a table of the temperatures taken in the case of a child who died from meningitis in April last.

| | Temperature of body. | Temperature of room. |
|---|---|---|
| Directly after death ... ... ... | 39·5° | — |
| 1 hour after ... ... ... ... | 36 | — |
| 5 hours after ... ... ... | 18 | 22° |
| 8 hours after ... ... ... | 14 | 19 |
| 11 hours after ... ... ... | 11 | 17 |
| 14 hours after ... ... ... | 11 | 14 |
| 17 hours after ... ... ... | 22 | 22 |

It will be seen from this table that five hours after death the temperature was lowered below that of the room; since at the end of 17 hours the corpse was in equilibrium with the temperature of the room where it was placed, it is clear that it follows the temperature of the surrounding atmosphere, and has no longer the invariable temperature of the living animal body; whence it follows that the thermometer furnishes, at least in this climate, a certain means of distinguishing real from apparent death.

### TRAMPS AND SMALL-POX.

Dr. Armstrong, Medical Officer of Health for Newcastle-upon-Tyne, calls attention to the important part played by tramps in the spread of infection. He sums up the results of queries sent by him to the medical officers of health of the 116 extra metropolitan urban districts with regard to the late rapid spread of small-pox throughout England, and concludes that the mischief and danger arising from the want of any control over the vagrant class are great, and demand, in the interests of the community, serious legislation. The answers to the queries addressed by Dr. Armstrong show that of the 63 urban districts invaded by small-pox, in no less than 37, or 59 per cent., the disease is known to have been first introduced by vagrants, and in nine of the other districts subsequent outbreaks of the infection were traced to the same source. A large majority of the medical officers of health consider (1) that vagrants should be restrained in their powers of carrying infection about the country, especially in epidemic times. (2) That they should be made to report their movements. (3) That they should, when requisite, be subject to detention, re-vaccination, and disinfection.

### OPHTHALMOLOGY IN RUSSIA.

The Society for Promoting the Welfare of the Blind in Russia, which is under the patronage of the Czarina, has arranged to send several young ophthalmic surgeons this summer in "flying columns" to various localities which are far removed from skilled attendance. They will do what they can toward the diminution of blindness, of which there is a great deal in Russia, both by treating eye diseases which if neglected might end in loss of sight, and also by operating on cases where it may be possible to restore sight by surgical means.

### A DRUNKARD'S MEMORY.

The *Quarterly Journal of Inebriety* has an article tending to show that the depositions of habitual inebriates are invariably worthless. "Inebriates always suffer from failure of memory, which takes on widely different forms and degrees. The inebriate may have the delusion that his memory is sound and clear, and will unconsciously supply the defects by imagination and stoutly affirm they are real. He may by a dim sort of intuition retain a conception of his conduct under certain conditions, especially if they are along automatic lines of previous acts. It may be seriously questioned if any inebriate ever has a sound clear memory of events that occur during the drink period, and whether interested or not, he is ever a safe witness of past events." It is concluded that the study of the memory in inebriety will open up a new field of facts, and tend to destroy many delusions with regard to the harmlessness of continual indulgence in "alcoholism."

